# Integration of bulk and single-cell RNA-seq data identifies a cellular senescence-related prognostic signature in liver hepatocellular carcinoma

**DOI:** 10.1007/s12672-026-05440-z

**Published:** 2026-06-10

**Authors:** Rentong Liu, Bing Li, Yiqian Luo, Xiuwei Yan, Yuanyuan Wang, Song Jiang, Chengjie Gan, Yiwei Cheng

**Affiliations:** 1 The 967th Hospital of the Joint Logistic Support Force of the Chinese People’s Liberation Army, Dalian, 116000 China; 2 Department of Neurosurgery, Cancer Center, Zhejiang Provincial People’s Hospital, Affiliated People’s Hospital, Hangzhou Medical College, Hangzhou, 310014 China

**Keywords:** Hepatocellular carcinoma, Cellular senescence, Prognostic signature, Tumor microenvironment

## Abstract

Liver hepatocellular carcinoma (LIHC) is a heterogeneous malignancy with poor prognosis, but the landscape of cellular senescence-related genes (CSRGs) in LIHC remains incompletely characterized. Here, we curated 167 CSRGs and performed integrative bulk and single-cell RNA-seq analyses using TCGA, ICGC, and TISCH datasets. Fifteen prognostic CSRGs were identified and stratified LIHC patients into two distinct molecular subtypes: Cluster 2 exhibited an immune-excluded phenotype with poor prognosis, whereas Cluster 1 represented an immune-desert phenotype. A CSRGs-based risk score was constructed and validated as an independent prognostic factor across cohorts. Single-cell analysis identified EEF1E1 as a key CSRG predominantly expressed in malignant hepatocytes, correlating with advanced clinicopathological features and immune infiltration. Functional experiments demonstrated that EEF1E1 knockdown significantly suppressed LIHC cell migration and invasion. Collectively, this study establishes a robust CSRGs-based prognostic signature. It also identifies EEF1E1 as a functionally relevant gene with oncogenic potential and a potential therapeutic target in LIHC.

**Supplementary Information:**

The online version contains supplementary material available at 10.1007/s12672-026-05440-z.

## Introduction

 Liver Hepatocellular Carcinoma (LIHC) is the sixth most common malignancy and the third leading cause of cancer-related mortality worldwide, accounting for approximately 780,000 deaths annually [1–3]. Despite advances in surgical resection, locoregional therapies, and systemic treatments such as tyrosine kinase inhibitors and immune checkpoint inhibitors, the prognosis of LIHC patients remains dismal, with a five-year survival rate of less than 20% [4, 5]. The high heterogeneity of LIHC at both the genomic and phenotypic levels poses a major challenge for precise risk stratification and individualized treatment [6–8]. Therefore, identifying reliable biomarkers and novel therapeutic targets that reflect the underlying biological heterogeneity is urgently needed to improve patient outcomes.

Cellular senescence is a fundamental biological process characterized by irreversible cell cycle arrest, which serves as a critical barrier against tumor initiation and progression [9, 10]. However, emerging evidence has revealed a paradoxical role of senescence in cancer. While oncogene-induced senescence can suppress tumorigenesis, the accumulation of senescent cells within the tumor microenvironment (TME) can paradoxically promote tumor growth, metastasis, and therapy resistance through the secretion of a complex mixture of pro-inflammatory cytokines, growth factors, and proteases, collectively termed the senescence-associated secretory phenotype (SASP) [11, 12]. The SASP can induce chronic inflammation, remodel the extracellular matrix, recruit immunosuppressive cells, and facilitate immune evasion, thereby creating a pro-tumorigenic niche [13, 14]. Given the dual nature of cellular senescence, the expression patterns and functional roles of senescence-related genes (CSRGs) in LIHC warrant comprehensive investigation.

Recent advances in high-throughput sequencing technologies have enabled the systematic characterization of the molecular landscape of LIHC [15]. However, most studies have focused on individual senescence-associated genes or pathways. A comprehensive analysis integrating bulk and single-cell RNA-seq data to identify CSRGs with prognostic and immunologic significance in LIHC is still lacking [16]. scRNA-seq offers a powerful approach to dissect cellular heterogeneity within the TME, allowing the identification of cell-type-specific expression patterns that are obscured in bulk tissue analyses [17]. By integrating bulk and single-cell data, it is possible to uncover previously unrecognized associations between senescence-related genes, immune cell infiltration, and clinical outcomes.

In this study, we performed a comprehensive integrative analysis to characterize the landscape of CSRGs in LIHC. Using transcriptomic data from The Cancer Genome Atlas (TCGA) and the International Cancer Genome Consortium (ICGC), we identified 15 prognostic CSRGs that were upregulated in LIHC tumor tissues. Unsupervised consensus clustering based on these genes stratified patients into two distinct molecular subtypes with divergent clinical outcomes and immune phenotypes. We further constructed and validated a CSRGs-based risk score that independently predicted overall survival and demonstrated strong associations with immune infiltration and immune checkpoint gene expression. Through single-cell resolution analysis, we identified EEF1E1 as a key CSRG specifically enriched in malignant hepatocytes and experimentally validated its pro‑migratory and pro‑invasive functions role in promoting cell migration and invasion. Collectively, our findings provide novel insights into the role of cellular senescence in LIHC and offer potential biomarkers for risk stratification and therapeutic targeting.

## Materials and methods

### Data collection and preprocessing

Transcriptomic profiles and corresponding clinical information of patients with LIHC were obtained from TCGA database (https://portal.gdc.cancer.gov). The LIHC dataset included 374 tumor samples and 50 adjacent normal tissue samples. FPKM values were converted to TPM for normalization. For external validation, the ICGC LIHC dataset was downloaded from the ICGC Data Portal (https://dcc.icgc.org). Samples with incomplete survival information or ambiguous histopathological diagnosis were excluded from further analysis.

### Identification of cellular senescence-related genes

A total of 167 CSRGs were curated from the Gene Ontology (GO) database using the following core accession terms: GO:0090398 (cellular senescence), GO:2,000,772 (regulation of cellular senescence), GO:2,000,773 (negative regulation of cellular senescence), and GO:2,000,774 (positive regulation of cellular senescence). These four terms were selected because they collectively represent the core cellular senescence process and its complete regulatory axis (positive and negative regulation), an approach widely adopted in previous senescence‑related prognostic studies.

### Differential expression analysis and prognostic gene screening

Differential expression analysis between LIHC tumor tissues and adjacent normal tissues was performed using the limma package in R [18]. Genes with |log₂FC| > 1 and a false discovery rate (FDR) < 0.05 were considered significantly upregulated. This threshold was selected based on standard practice in cancer transcriptomics to identify biologically meaningful expression differences while controlling for multiple testing, as recommended for TCGA‑based analyses [18]. In the context of LIHC, this cutoff has been widely applied to capture genes with moderate‑to‑large effect sizes. Moreover, because cellular senescence‑related genes often exhibit modest upregulation in tumors, a less stringent fold‑change threshold (|log₂FC| > 1) was used to avoid missing potentially relevant genes.

Univariate Cox regression analysis was applied to identify CSRGs significantly associated with overall survival (OS) in the TCGA cohort. Genes with a P value < 0.05 were considered prognostic.

### Consensus clustering for molecular subtype identification

Based on the expression profiles of the overlapping prognostic CSRGs, unsupervised consensus clustering was performed using the ConsensusClusterPlus R package (version 1.74.0) [19]. The following parameters were used: reps = 100, pItem = 0.8, pFeature = 1, clusterAlg = “km”, distance = “euclidean”, innerLinkage = “complete”, and finalLinkage = “complete”. A total of 1,000 iterations with an 80% sampling rate were conducted to ensure stability. The optimal number of clusters (k) was determined by evaluating the empirical cumulative distribution function (CDF) curves, the relative change in the area under the CDF curve, and the consensus matrix. Patients were stratified into two distinct molecular subtypes (Cluster 1 and Cluster 2).

### Immune microenvironment analysis

The immune landscape of LIHC patients was evaluated using multiple algorithms. The relative proportions of immune cell infiltration were estimated using the TIMER [20], MCP-counter [21], and xCell [22] algorithms implemented via the IOBR package (version 2.2.0) [23]. The activity of the cancer-immunity cycle was assessed using the Tumor Immune Estimation Resource (TIP) database (http://biocc.hrbmu.edu.cn/TIP/index.jsp), which visualizes anti-tumor immune responses across seven sequential steps: release of cancer cell antigens (Step 1), cancer antigen presentation (Step 2), priming and activation (Step 3), trafficking of immune cells to tumors (Step 4), infiltration of immune cells into tumors (Step 5), recognition of cancer cells by T cells (Step 6), and killing of cancer cells (Step 7) [24].

### Construction and validation of a prognostic risk signature

Least absolute shrinkage and selection operator (LASSO) penalized regression was performed using the glmnet R package (version 4.1–8) to select the most informative CSRGs and construct a risk score model [25]. Ten-fold cross-validation was applied to determine the optimal tuning parameter (λ). The risk score for each patient was calculated as follows:

$$\:Risk\:score\:={\sum\:}_{i=1}^{n}Coef\:\left({X}_{i}\right)*\:Exp\:\left({X}_{i}\right)$$

where Coef (*X*_i_) represents the regression coefficient of each selected gene, and Exp (*X*_i_) represents its expression level. represents its expression level. The genes and their corresponding coefficients are also provided in Table S1. Patients were divided into high-risk and low-risk groups based on the median risk score. The predictive performance of the risk signature was evaluated using Kaplan-Meier survival curves with log-rank tests and time-dependent receiver operating characteristic (ROC) curves with the area under the curve (AUC) calculated using the timeROC R package (version 0.4.1). Principal component analysis (PCA) and t-distributed stochastic neighbor embedding (t-SNE) were performed to visualize the clustering of high- and low-risk groups.

### Independent prognostic analysis and nomogram construction

Univariate and multivariate Cox regression analyses were performed to assess whether the risk score served as an independent prognostic factor, adjusting for clinicopathological variables including age, gender, histological grade, and pathological stage. A nomogram was constructed integrating the risk score and significant clinicopathological features to predict 1-, 3-, and 5-year overall survival [26]. Calibration curves were generated to evaluate the agreement between predicted and observed survival probabilities.

### Single-cell RNA-seq data analysis

Single-cell RNA-sequencing data of LIHC samples were obtained from the Tumor Immune Single-Cell Hub (TISCH) database (http://tisch.comp-genomics.org). The LIHC dataset with accession number GSE166635 was retrieved from the TISCH portal [27]. TISCH provides comprehensive single‑cell transcriptomic profiles with standardized processing, including quality control, batch effect removal, dimensionality reduction, clustering, and cell type annotation. For this study, we directly accessed the pre‑processed UMAP projections and cell type annotations from TISCH. The expression distribution of the 15 prognostic CSRGs across different cell types was visualized using the built‑in plotting tools or downloaded from the TISCH web interface.

### Cell culture and siRNA transfection

Human LIHC cell lines HepG-2 and HuH-7 were obtained from the Cell Bank of the Chinese Academy of Sciences (Shanghai, China) and cultured in DMEM supplemented with 10% FBS and 1% penicillin-streptomycin at 37 °C in a 5% CO₂ atmosphere. Small interfering RNA (siRNA) targeting EEF1E1 and negative control siRNA were synthesized and transfected using Lipofectamine 3000 reagent according to the manufacturer’s instructions. Knockdown efficiency was confirmed by RT‑qPCR and western blot analysis 48 h post-transfection.

### RNA extraction and quantitative real-time PCR (RT‑qPCR)

Total RNA was extracted from cell lines and tissue samples using TRIzol reagent. Reverse transcription was performed using the PrimeScript RT Reagent Kit. RT‑qPCR was conducted using SYBR Green Master Mix on a real-time PCR system. Relative gene expression levels were calculated using the 2^⁻ΔΔCt^ method, with GAPDH as the internal control. All reactions were performed in triplicate.

### Western blot analysis

Cells and tissues were lysed in RIPA buffer containing protease and phosphatase inhibitors. Protein concentrations were quantified using the BCA assay. Equal amounts of protein were separated by SDS-PAGE and transferred to PVDF membranes. Membranes were blocked with 5% non-fat milk and incubated overnight at 4 °C with primary antibodies against EEF1E1 (1:1000,10805-1-AP, Proteintech) and β-tubulin (1:4000, AF1216, Beyotime). After incubation with HRP-conjugated secondary antibodies, protein bands were visualized using enhanced chemiluminescence substrate.

### Wound healing and Transwell invasion assays

For wound healing assays, cells were seeded in six-well plates and grown to confluence. A sterile pipette tip was used to create a linear scratch. Wound closure was monitored and photographed at 0 and 24 h. The percentage of wound closure was quantified using ImageJ software. For Transwell invasion assays, cells were suspended in serum-free medium and seeded into the upper chamber of Matrigel-coated inserts. The lower chamber was filled with medium containing 10% FBS as a chemoattractant. After 24 h, non‑invading cells were removed, and invading cells were fixed, stained with crystal violet, and counted in five random fields per well.

### Statistical analysis

All statistical analyses were performed using R software (version 4.5.2). Comparisons between two groups were performed using the Wilcoxon rank-sum test. Comparisons among multiple groups were performed using the Kruskal-Wallis test. Categorical variables were compared using the chi‑square test or Fisher’s exact test. Survival curves were estimated using the Kaplan‑Meier method and compared using the log‑rank test. A P value < 0.05 was considered statistically significant.: Significance levels: *P < 0.05, **P < 0.01, ***P < 0.001.

## Results

### Identification of cellular senescence-related molecular subtypes in LIHC 

**Fig. 1:**
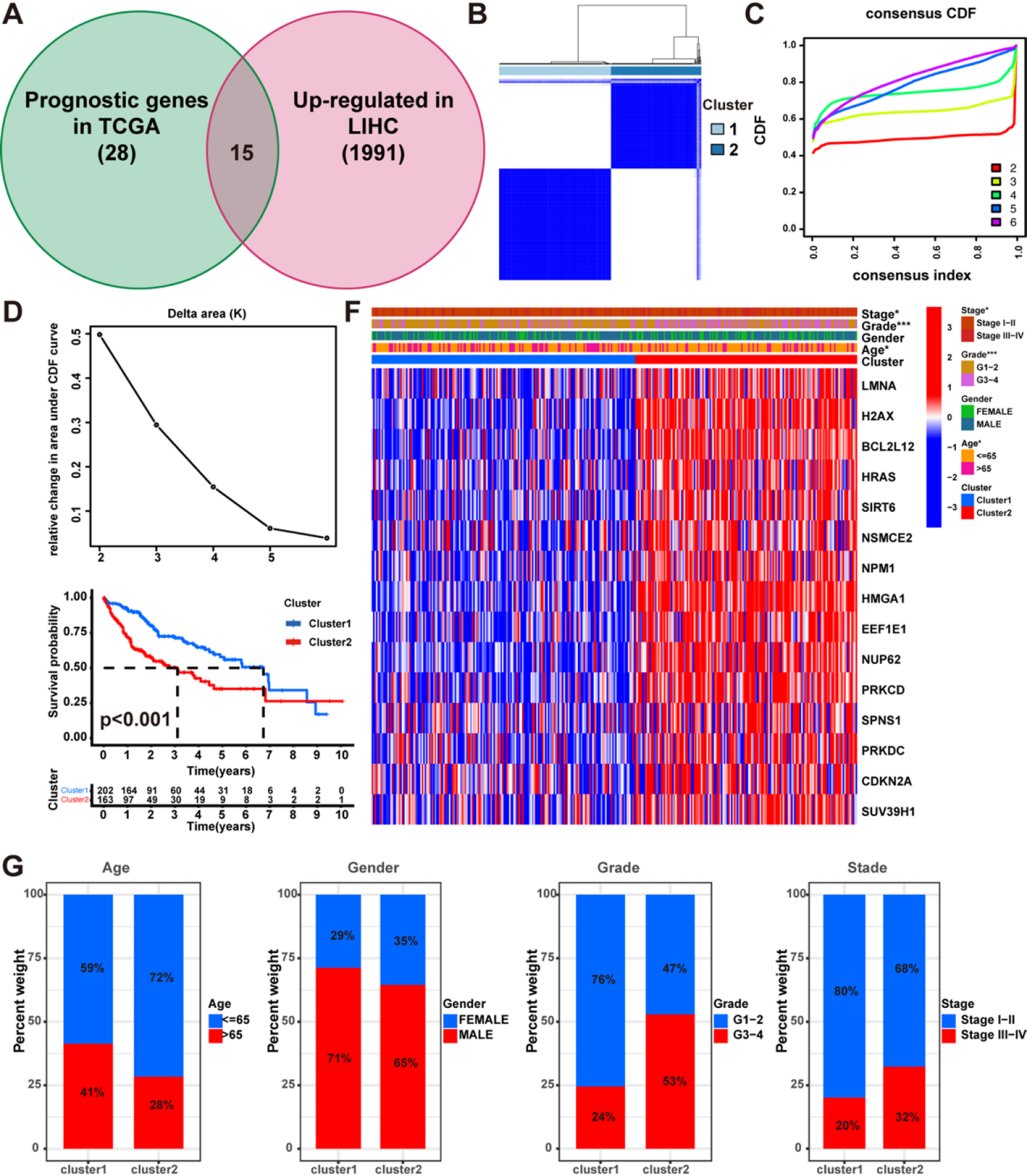
Identification of CSRGs‑based molecular subtypes in LIHC. **A** Venn diagram showing the overlap between upregulated genes in tumor tissues (|logFC| > 1) and prognosis‑associated CSRGs identified by univariate Cox regression analysis. A total of 15 overlapping genes were selected for further analysis (**B–D**) Consensus clustering of LIHC patients based on the expression profiles of the 15 prognostic CSRGs. **B** Empirical CDF curves for k = 2 to 10. **C** Relative change in the area under the CDF curve, indicating that k = 2 represents the optimal clustering solution. **D** Consensus matrix for k = 2, clearly delineating two distinct molecular subtypes. **E** Kaplan‑Meier survival curves for patients in Cluster 1 and Cluster 2 in the TCGA cohort. **F** Heatmap displaying the expression patterns of the 15 prognostic CSRGs across the two subtypes. Clinical annotations, including histological grade and pathological stage, are shown at the top. **G** Bar plots illustrating the distribution of Clinical pathological features between Cluster 1 and Cluster 2

A total of 167 CSRGs were initially retrieved from the GO database based on the following accession terms: GO:0090398, GO:2,000,772, GO:2,000,773, and GO:2,000,774. Univariate Cox regression analysis identified 43 CSRGs significantly associated with overall survival in patients with LIHC. Concurrently, differential expression analysis between LIHC and adjacent normal tissues using the limma package (|log₂FC| > 1, FDR < 0.05) yielded 2,016 upregulated genes. Intersection of these two gene sets revealed 15 prognostic CSRGs that were upregulated in tumor tissues (Fig. 1A).

To explore the underlying heterogeneity among LIHC patients, we performed unsupervised consensus clustering based on the expression profiles of these 15 genes. Analysis of the CDF and consensus matrices indicated that k = 2 represented the optimal clustering solution, allowing the stratification of patients into two distinct molecular subtypes, designated Cluster 1 and Cluster 2 (Fig. 1B and D).

Significant differences in clinical outcomes were observed between the two subtypes. Kaplan–Meier survival analysis demonstrated that patients in Cluster 1 had markedly longer overall survival compared to those in Cluster 2 (Fig. 1E). We further characterized the association between the identified subtypes, CSRG expression, and clinicopathological features. Heatmaps revealed distinct expression patterns of the 15 prognostic CSRGs between Cluster 1 and Cluster 2, accompanied by distinct distributions of clinical annotations (Fig. 1F). Notably, Cluster 2 was associated with a more aggressive clinical phenotype. In the TCGA cohort, patients in Cluster 2 exhibited significantly higher proportions of advanced histological grade and pathological stage compared to those in Cluster 1 (Fig. 1G).

###  Immunogenomic landscape of CSRGs-based molecular subtypes in LIHC

**Fig. 2:**
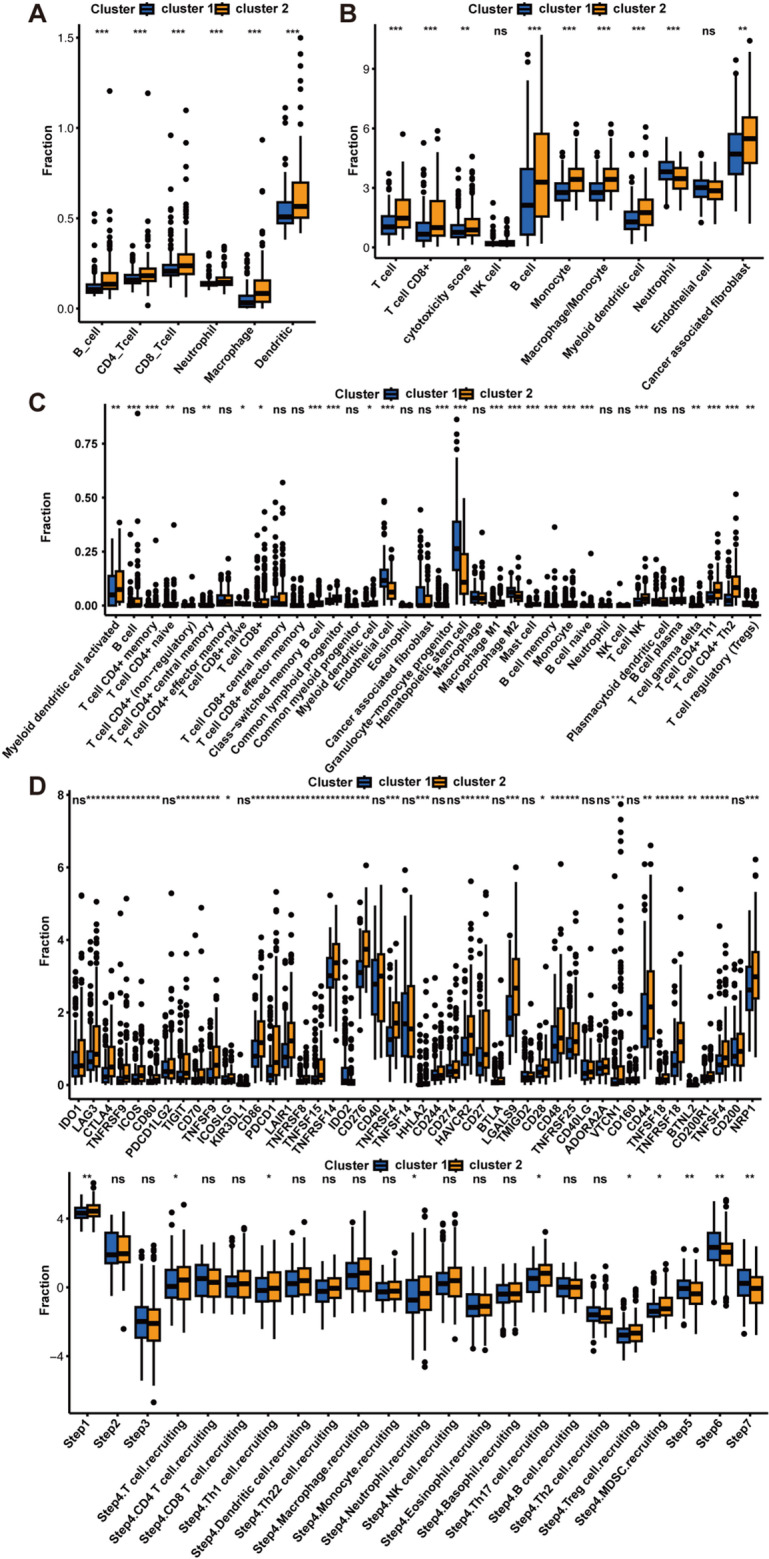
Immunogenomic landscape of CSRGs‑based molecular subtypes in LIHC. **A–C** Immune cell infiltration abundance in Cluster 1 and Cluster 2 estimated by three independent algorithms: **A** TIMER, **B** MCP‑counter, and **C** xCell. **D** Expression levels of canonical immune checkpoint genes, including PD‑L1, PD‑1, and CTLA‑4, in the two subtypes. Most checkpoint genes were significantly upregulated in Cluster 2 relative to Cluster 1. **E** Activity profiling of the cancer‑immunity cycle steps using the TIP database (*P < 0.05, **P < 0.01, ***P < 0.001)

To further characterize the biological distinction between the two subtypes, we investigated the landscape of the TME. Using the IOBR package, we estimated immune cell infiltration with three independent algorithms: TIMER, MCP-counter, and xCell. Notably, Cluster 2 exhibited significantly higher enrichment scores for the majority of immune cell types compared to Cluster 1, suggesting a more inflamed, albeit potentially immunosuppressive, TIME (Fig. 2A–C). Given the pivotal role of immune checkpoint pathways in tumor immune evasion, we next examined the expression profiles of canonical immune checkpoint genes between the two clusters. Consistent with the immune infiltration patterns, most immune checkpoint genes, including PD-L1, PDCD1, and CTLA-4, were significantly upregulated in Cluster 2 relative to Cluster 1 (Fig. 2D).

To gain deeper insight into the dynamic processes underlying these immune phenotypes, we leveraged the TIP database to assess the activity of distinct immune cell populations across the cancer-immunity cycle. Intriguingly, Cluster 1 exhibited significantly higher activity in steps 5, 6, and 7, which correspond to immune cell infiltration, killing of tumor cells, and release of tumor antigens, respectively. In contrast, Cluster 2 showed enhanced enrichment in steps 1 and 4, representing antigen release and trafficking of immune cells to the tumor site (Fig. 2E). These divergent patterns suggest that while Cluster 2 displays a globally higher abundance of immune cells, the effective execution of anti-tumor immunity may be arrested at intermediate steps. Based on these findings, we propose that Cluster 2 corresponds to an immune-excluded phenotype, characterized by immune cell accumulation at the periphery without effective tumor infiltration, whereas Cluster 1 represents an immune-desert phenotype, marked by minimal immune engagement across the majority of the cancer-immunity cycle [28].

### Construction and validation of a CSRGs-based prognostic risk signature

**Fig. 3:**
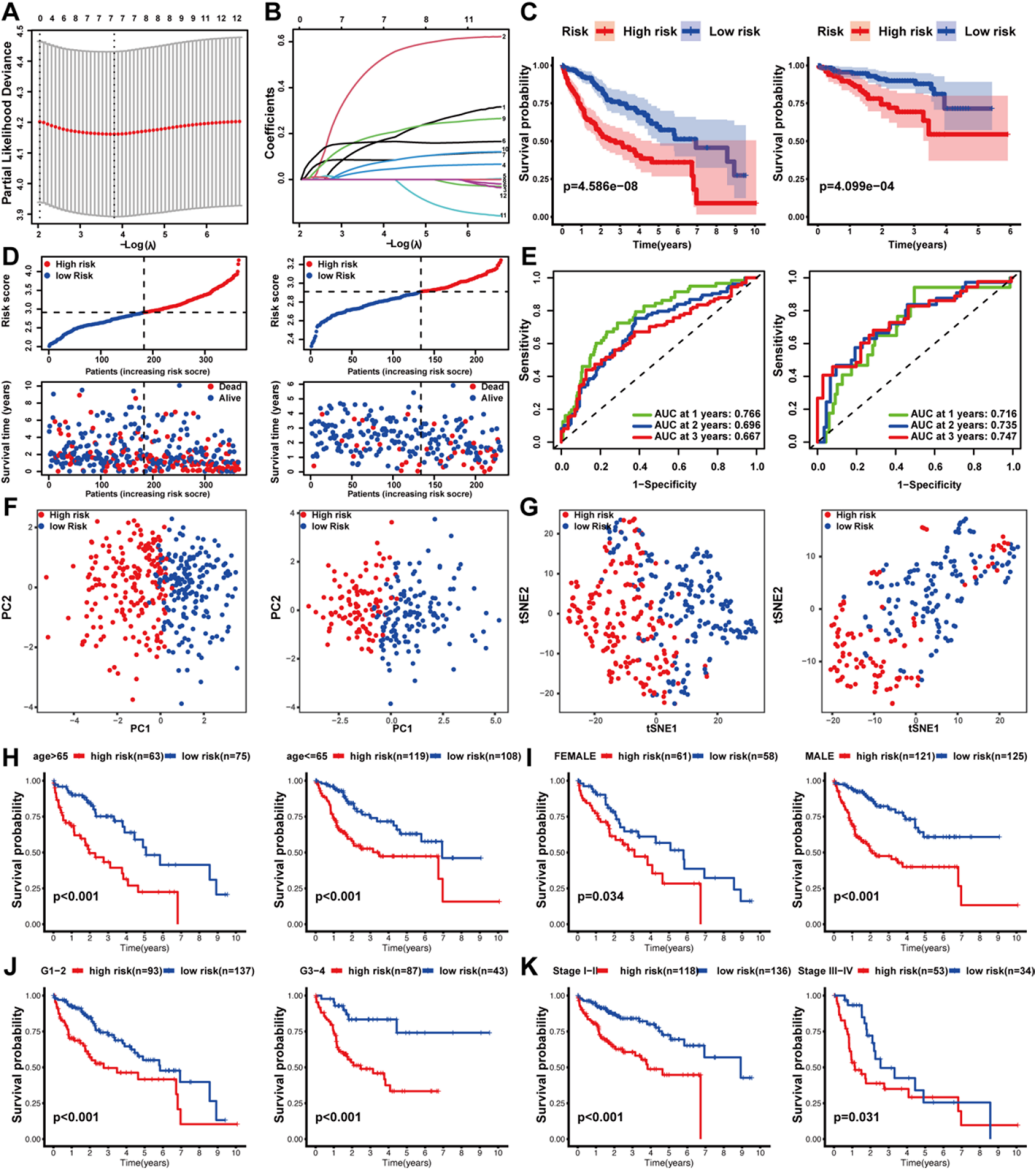
Construction and validation of a CSRGs‑based prognostic risk signature in LIHC. **A**, **B** LASSO penalized regression analysis for feature selection. **A** Partial likelihood deviance curves showing the optimal tuning parameter (λ) selection via ten‑fold cross‑validation. **B** Coefficient profiles of the candidate CSRGs as a function of the log(λ) sequence. **C** Kaplan‑Meier survival curves comparing overall survival between high‑risk and low‑risk groups in the TCGA (left) and ICGC (right) cohorts. **D** Distribution of risk scores (upper panel) and survival status (lower panel) in the TCGA (left) and ICGC (right) cohorts. **E** Time‑dependent receiver operating characteristic (ROC) curves for the risk score predicting 1‑, 3‑, and 5‑year overall survival in the TCGA (left) and ICGC (right) cohorts. **F**, **G** Dimensionality reduction analyses showing clear separation between high‑risk and low‑risk groups. **F** PCA. **G** t‑SNE. **H–K** Subgroup survival analyses stratified by clinicopathological features n the TCGA dataset. In each subgroup, patients in the low‑risk group consistently demonstrated superior survival outcomes compared with those in the high‑risk group, including across different **H **Age, **I** Gender, **J **histological grade, and **K** pathological stages

To develop a quantitative prognostic model based on the identified CSRGs, we performed LASSO penalized regression analysis using LIHC datasets from TCGA and the ICGC. The final LASSO model retained seven CSRGs with non‑zero coefficients, as summarized in Supplementary Table S1. A risk score was subsequently constructed for each patient, enabling stratification into high- and low-risk groups (Fig. 3A and B). Kaplan–Meier survival analysis revealed that patients in the low-risk group had significantly prolonged overall survival compared to those in the high-risk group across both cohorts (both *P* < 0.001; Fig. 3C). The distribution of risk scores and survival status further illustrated that patients with higher risk scores experienced shorter survival times and increased mortality (Fig. 3D). Time-dependent ROC analysis demonstrated favorable predictive performance of the risk score. In the TCGA cohort, the AUC values for 1-, 3-, and 5-year overall survival were 0.766, 0.696, and 0.667, respectively. Comparable performance was observed in the ICGC cohort, with AUC values of 0.716, 0.735, and 0.747 for the respective time points (Fig. 3E). Dimensionality reduction analyses, including PCA and t-SNE, showed clear separation between high- and low-risk groups, indicating robust clustering based on the CSRGs-derived risk score (Fig. 3F and G). Subgroup survival analyses stratified by various clinicopathological features consistently showed that patients in the low-risk group experienced superior survival outcomes compared to those in the high-risk group across different clinical strata, including histological grade, pathological stage, and other key variables (Fig. 3H and K, Figure S1A-C). Collectively, these findings validate the prognostic utility of the CSRGs-based risk signature across independent cohorts and clinical subgroups.

**Fig. 4:**
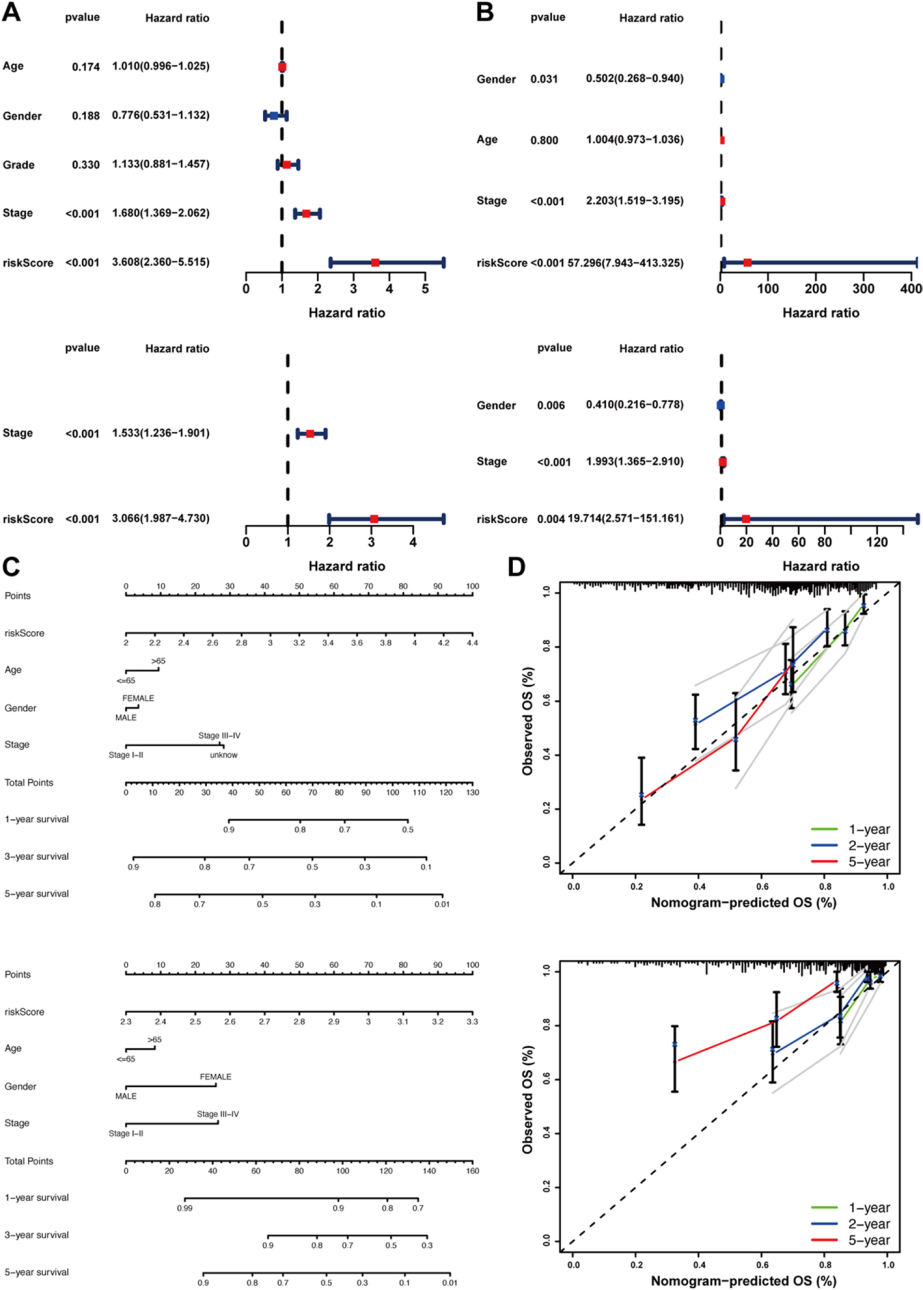
Independent prognostic value and clinical utility of the CSRGs‑based risk signature.**A**, **B** Forest plots illustrating the results of univariate and multivariate Cox regression analyses for overall survival in LIHC patients. In the TCGA cohort, the risk score was identified as an independent prognostic factor in both models (univariate: HR = 1.680, 95% CI = 1.369–2.062, *P* < 0.001; multivariate: HR = 2.203, 95% CI = 1.519–3.195, *P* < 0.001). Consistent results were observed in the ICGC cohort, where the risk score also demonstrated independent prognostic significance (univariate: HR = 1.533, 95% CI = 1.236–1.901, *P* < 0.001; multivariate: HR = 1.631, 95% CI = 1.298–2.050, *P* < 0.001). **C** Nomogram constructed integrating the risk score with key clinicopathological variables (age, gender, histological grade, and pathological stage) for predicting 1‑, 3‑, and 5‑year overall survival probability in the TCGA (left) and ICGC (right) cohort. **D** Calibration curves demonstrating optimal agreement between predicted and observed survival probabilities at 1, 3, and 5 years. Curves for both the TCGA (left) and ICGC (right) cohorts are shown, confirming robust predictive accuracy of the nomogram across independent datasets

To evaluate the clinical utility of the LASSO-derived risk score, we performed univariate and multivariate Cox regression analyses. In both models, the risk score emerged as an independent prognostic factor for overall survival in LIHC patients (Fig. 4A, B). To facilitate clinical translation, a nomogram integrating the risk score with key clinicopathological features was constructed, which demonstrated robust predictive performance as evidenced by well-calibrated calibration curves (Fig. 4C, D).

### Immune landscape in high- and low-risk groups

**Fig. 5:**
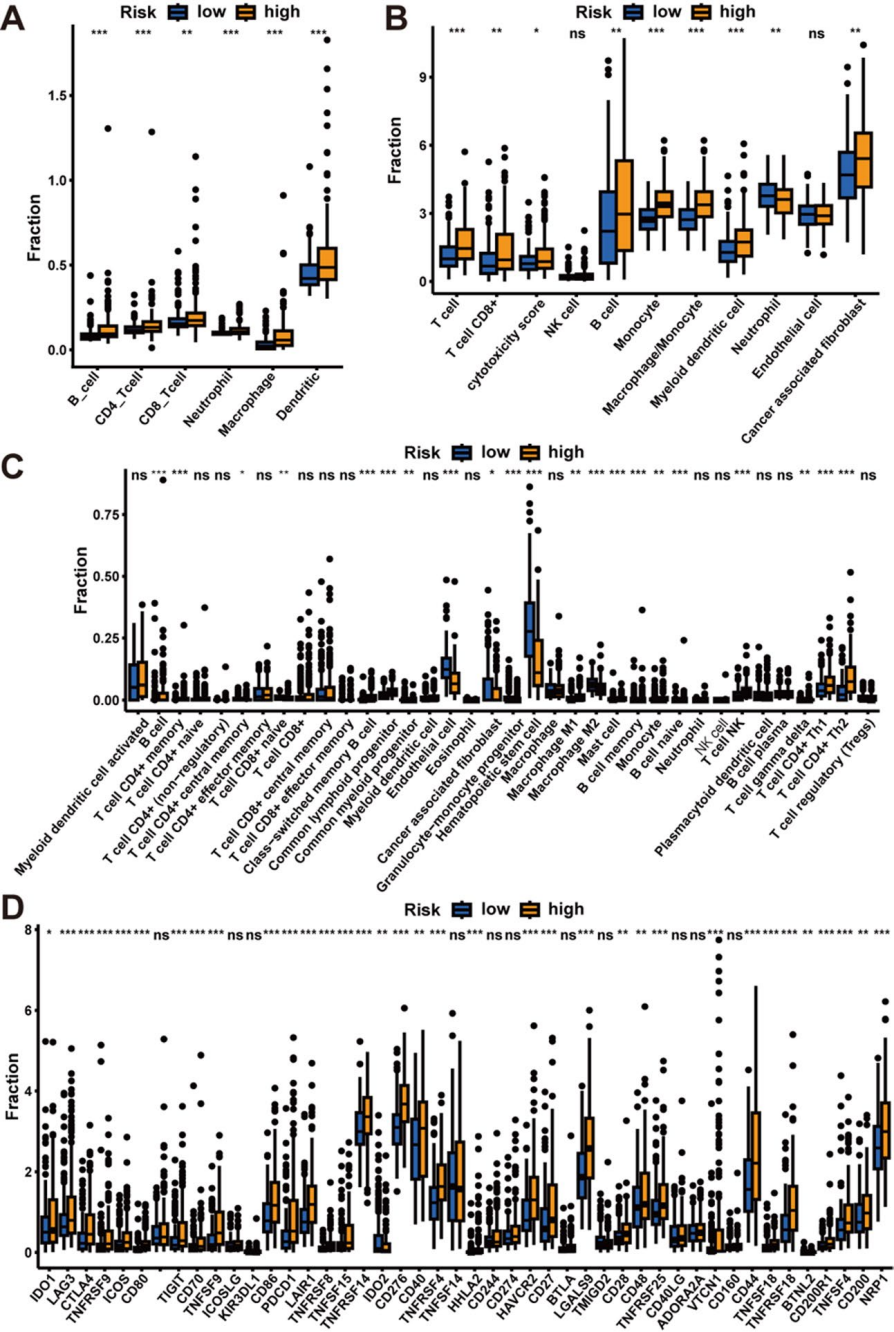
Immune landscape between high‑ and low‑risk groups in the TCGA cohort. **A–C **Bar plots showing the infiltration abundance of diverse immune cell types estimated by **A** TIMER, **B** MCP‑counter, and **C** xCell algorithms in the TCGA cohort **D** Expression levels of canonical immune checkpoint genes, in high‑risk versus low‑risk groups (*P < 0.05, **P < 0.01, ***P < 0.001)

To further characterize the immune microenvironment associated with the CSRGs‑based risk signature, we evaluated immune cell infiltration and immune checkpoint gene expression between the high‑ and low‑risk groups. Using three independent algorithms—TIMER, MCP‑counter, and xCell—implemented, we observed that patients in the high‑risk group exhibited significantly higher enrichment scores for the majority of immune cell types compared with those in the low‑risk group (Fig. 5A–C; Figure S2A–C). Consistent with this pattern, the expression levels of canonical immune checkpoint genes, including PD‑L1, PD‑1, and CTLA‑4, were markedly upregulated in the high‑risk group relative to the low‑risk group (Fig. 5D; Figure S3D). These findings indicate that the risk score not only captures prognostic heterogeneity but also reflects distinct immune phenotypes, with the high‑risk group displaying a more inflamed yet potentially immunosuppressive tumor microenvironment.

### Identification of EEF1E1 as a key CSRG in LIHC

**Fig. 6:**
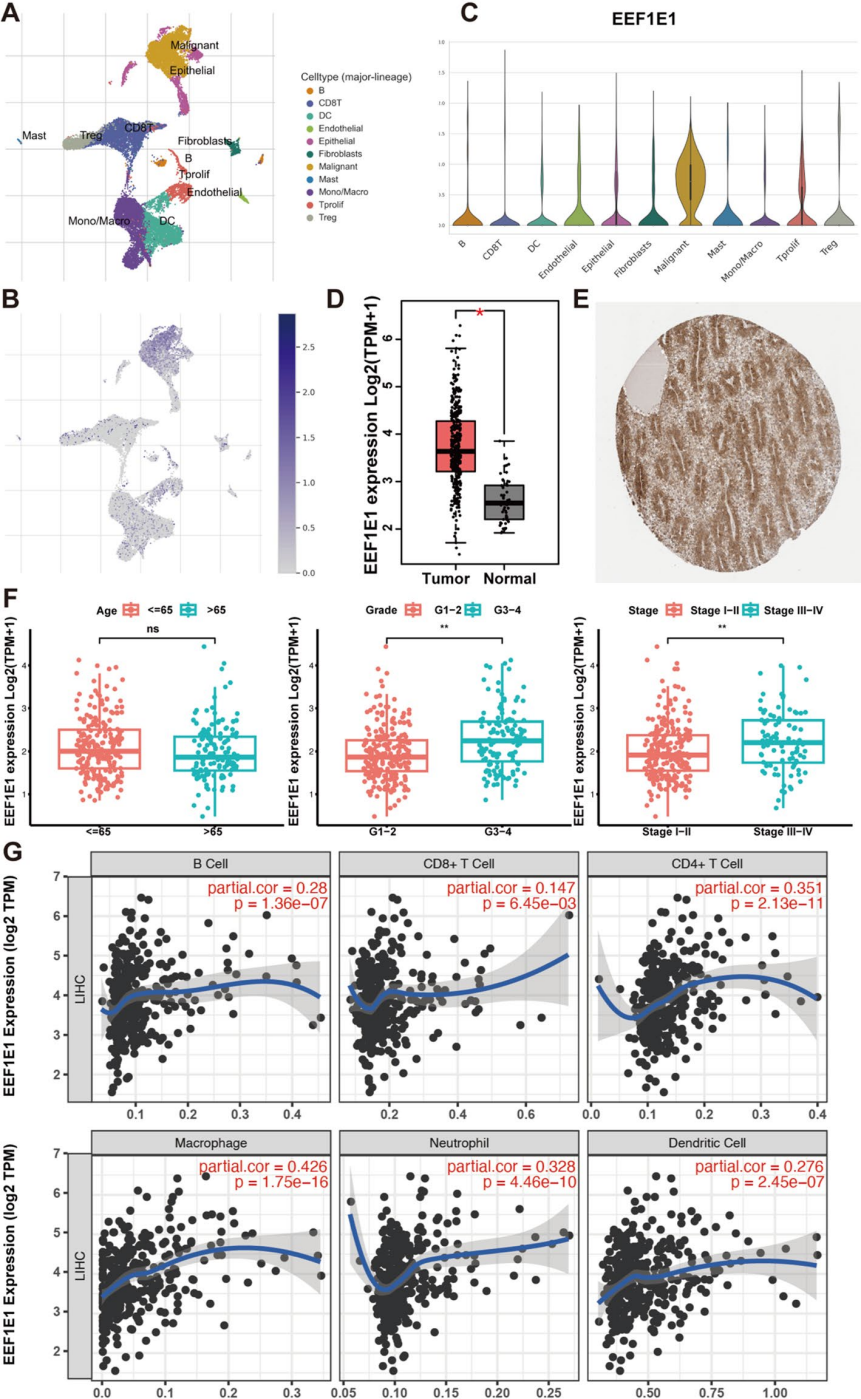
Identification and validation of EEF1E1 as a key CSRG in LIHC. **A** UMAP plot derived from single‑cell RNA‑sequencing data of the GSE166635 dataset, illustrating distinct cellular clusters within the LIHC tumor microenvironment. **B** UMAP plot showing the expression distribution of EEF1E1 projected onto the same dimensional reduction space (right). **C** Violin plots showing the expression distribution of EEF1E1 across distinct cell types in the GSE166635 single‑cell dataset. **D** Box plots showing the expression levels of EEF1E1 in LIHC tumor tissues compared with adjacent normal tissues. **E** The protein expression of EEF1E1 in HPA dataset. **F **The association between EEF1E1 expression and clinical pathological features in the TCGA dataset. **G** Correlation analysis between EEF1E1 expression and immune cell infiltration levels using the TIMER database (*P < 0.05, **P < 0.01, ***P < 0.001)

To further pinpoint the most functionally relevant CSRGs within the prognostic signature, we analyzed single‑cell RNA‑sequencing data from the GSE166635 dataset obtained from the TISCH database. Dimensionality reduction via UMAP revealed distinct cellular clusters within the LIHC tumor microenvironment (Fig. 6A). Violin plots based on TISCH‑provided annotations showed that EEF1E1 was highly enriched in malignant hepatocytes compared with other cell types (immune and stromal populations) (Fig. 6B, C; Figure S3A–C). This was observed among the seven prognostic CSRGs. The remaining six genes (HRAS, SPNS1, CDKN2A, PRKCD, NPM1, PRKDC) either exhibited broad expression across multiple cell types or were more abundant in immune/stromal compartments. This unique cell‑type specificity led us to prioritize EEF1E1 for further functional validation.

We next validated the expression of EEF1E1 at the transcriptional level using the GEPIA database. Consistent with the single‑cell findings, EEF1E1 expression was significantly upregulated in LIHC tumor tissues compared with adjacent normal tissues (Fig. 6D). Immunohistochemical analysis from the Human Protein Atlas (HPA) database further confirmed elevated EEF1E1 protein expression in LIHC tumor samples (Fig. 6E). To explore the clinical relevance of EEF1E1, we analyzed its association with clinicopathological features. Notably, high EEF1E1 expression was significantly correlated with advanced histological grade and pathological stage, indicating its potential role in tumor aggressiveness (Fig. 6F; Figure S3D).

Given the established link between EEF1E1 and immune infiltration patterns observed in the risk model, we further investigated its correlation with immune cell infiltration using the TIMER database. EEF1E1 expression showed significant positive correlations with the infiltration levels of multiple immune cell types, including B cells, CD8⁺ T cells, CD4⁺ T cells, macrophages, neutrophils, and dendritic cells (Fig. 6G). These findings suggest that EEF1E1 may contribute to the immune‑modulatory landscape of LIHC. Importantly, the classification of EEF1E1 as a CSRG is derived from Gene Ontology annotations; whether its pro‑migratory and pro‑invasive functions are directly mediated through cellular senescence pathways or the SASP remains to be determined.

### Functional validation of EEF1E1 in LIHC cell lines

**Fig. 7:**
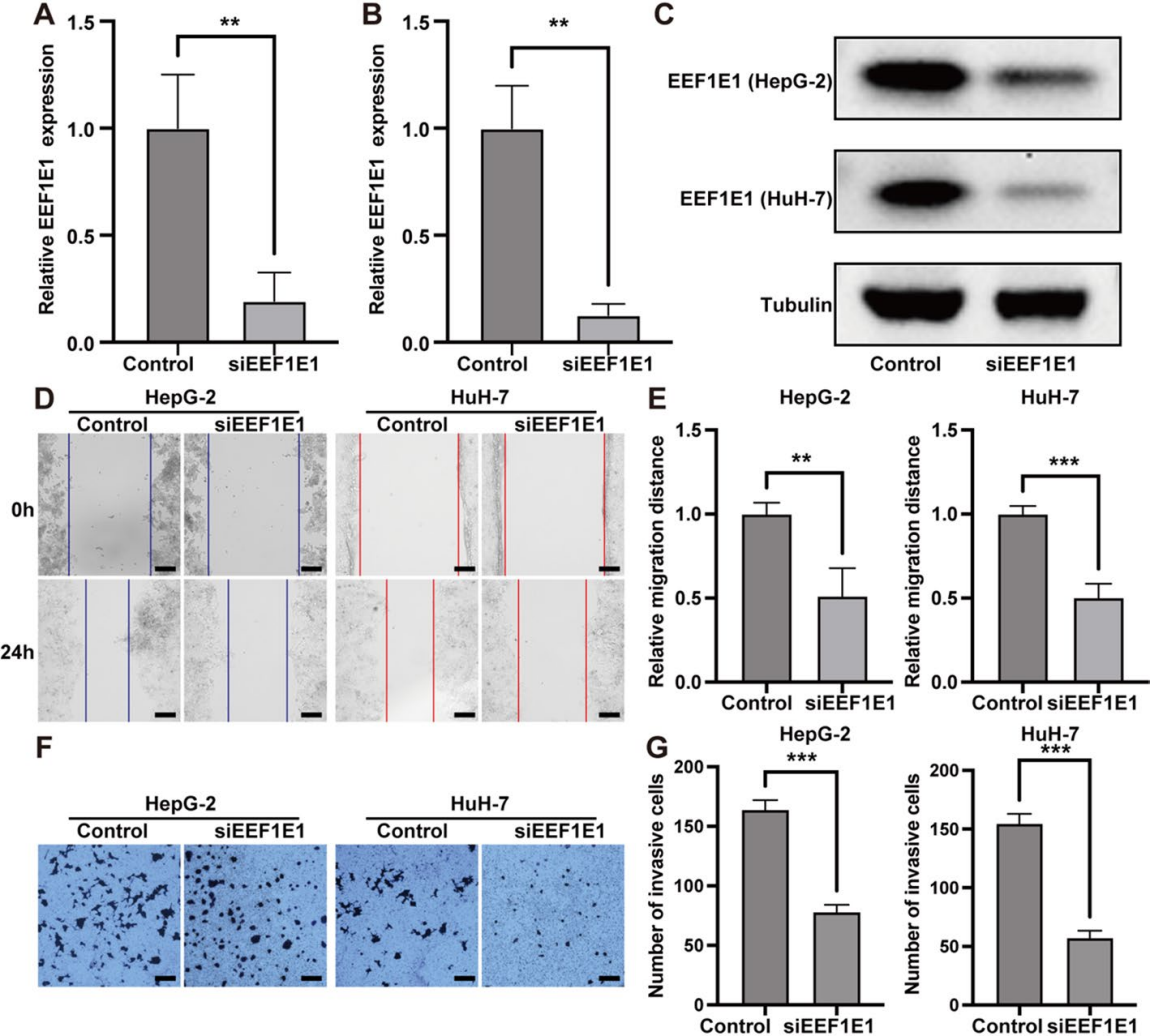
EEF1E1 knockdown suppresses migration and invasion of LIHC cells in vitro. **A–B** Validation of EEF1E1 knockdown efficiency in HepG‑2 and HuH‑7 cells. **A**, **B** RT‑qPCR analysis showing significantly reduced EEF1E1 mRNA expression following transfection with siEEF1E1 compared with control siRNA. **C** Western blot analysis confirming decreased EEF1E1 protein expression in both cell lines after siRNA‑mediated knockdown. **D**,** E** Wound healing assays assessing cell migration capacity. Representative images. **D** and quantitative analysis. E demonstrate that EEF1E1 knockdown significantly impaired the migratory ability of HepG‑2 and HuH‑7 cells compared with control groups. Scale bar: 200 μm. **F**, **G** Transwell assays evaluating cell invasion capacity. Representative images. F and quantitative analysis. **G** show that downregulation of EEF1E1 markedly reduced the invasive ability of both cell lines. Scale bar: 200 μm. All in vitro experiments (including RT‑qPCR, western blot, wound healing, and Transwell assays) were performed in three independent biological replicates. Comparisons between two groups (siControl vs. siEEF1E1) were performed using two‑sided Student’s t‑test. Data are presented as mean ± SD. Significance levels are indicated as *P < 0.05, **P < 0.01, ***P < 0.001

Given the consistent association of EEF1E1 with poor prognosis, immune infiltration, and specific enrichment in malignant hepatocytes, we further investigated its functional role in LIHC through loss‑of‑function experiments. siRNA‑mediated knockdown of EEF1E1 was performed in both HepG‑2 and HuH‑7 cell lines. The efficiency of knockdown was confirmed by RT‑qPCR and western blot analysis, demonstrating significant reduction of EEF1E1 at both the mRNA and protein levels in siEEF1E1‑treated cells compared with controls (Fig. 7A–C). Functional assays revealed that EEF1E1 silencing markedly suppressed the migratory capacity of LIHC cells, as evidenced by wound healing assays (Fig. 7D, E). Similarly, Transwell invasion assays demonstrated that knockdown of EEF1E1 significantly attenuated the invasive ability of both HepG‑2 and HuH‑7 cells (Fig. 7F, G). Collectively, these findings suggest that EEF1E1 promotes cell migration and invasion in LIHC, supporting its role as a functionally relevant gene promoting cell migration and invasion within the CSRGs‑based prognostic signature.

## Discussion

In the present study, we comprehensively characterized the landscape of CSRGs in LIHC and constructed a robust prognostic signature with potential clinical utility. By integrating bulk transcriptomic and single-cell RNA-sequencing data from multiple independent cohorts, we identified two distinct molecular subtypes (Cluster 1 and Cluster 2) with divergent prognostic outcomes and immune phenotypes. Furthermore, we developed and validated a CSRGs-based risk score that independently predicted overall survival and demonstrated significant associations with tumor immune infiltration and immune checkpoint gene expression. Notably, through single-cell resolution analysis, we pinpointed EEF1E1 as a key CSRG specifically enriched in malignant hepatocytes, and functional experiments confirmed its pro‑migratory and pro‑invasive functions in promoting LIHC cell migration and invasion. Collectively, our findings provide novel insights into the role of cellular senescence in LIHC and offer a potential biomarker and therapeutic target for this devastating disease.

Cellular senescence is a complex biological process characterized by irreversible cell cycle arrest, which has been implicated in both tumor suppression and tumor promotion depending on the context [29, 30]. While the tumor-suppressive functions of senescence are well established, accumulating evidence suggests that senescent cells can acquire a SASP that promotes chronic inflammation, immune evasion, and tumor progression [31, 32]. In hepatocellular carcinoma, the role of senescence-related genes remains incompletely understood, and systematic characterization of CSRGs in this malignancy is lacking. Our study addresses this gap by providing a comprehensive analysis of CSRGs in LIHC and revealing their prognostic and immunologic significance. Notably, conflicting evidence exists regarding the role of senescence in LIHC. CD4⁺ T‑cell‑mediated clearance of senescent pre‑malignant hepatocytes (senescence surveillance) suppresses LIHC development, representing the anti‑tumorigenic arm of senescence [33]. In contrast, NFATc1 amplifies the SASP to promote LIHC progression, independent of growth arrest, illustrating its pro‑tumorigenic potential [34]. Our findings of an immune‑excluded phenotype in Cluster 2, which exhibits high expression of SASP‑related CSRGs, align with the latter, pro‑tumorigenic facet of this duality.

Through unsupervised consensus clustering based on 15 prognostic CSRGs, we stratified LIHC patients into two distinct molecular subtypes with markedly different clinical outcomes. Cluster 1 was characterized by favorable prognosis, whereas Cluster 2 was associated with aggressive clinicopathological features and poor survival. These findings align with previous studies demonstrating that aberrant expression of senescence-associated genes correlates with tumor progression and therapy resistance in various cancers [35, 36]. Notably, the two subtypes exhibited distinct immune landscapes: Cluster 2 displayed higher overall immune cell infiltration but paradoxically showed impaired effector functions in the later stages of the cancer-immunity cycle. This pattern is reminiscent of the immune-excluded phenotype, in which immune cells accumulate at the tumor periphery but fail to infiltrate the tumor parenchyma and exert effective anti-tumor responses [28]. In contrast, Cluster 1 exhibited an immune-desert phenotype with minimal immune cell presence. These observations underscore the complex interplay between cellular senescence and immune modulation, suggesting that CSRGs may orchestrate immune evasion mechanisms that contribute to poor outcomes in LIHC.

The prognostic risk signature we constructed based on CSRGs demonstrated excellent predictive performance across both TCGA and ICGC validation cohorts, with AUC values exceeding 0.70 for 1-, 3-, and 5-year survival. I Importantly, the risk score remained an independent prognostic factor after adjusting for conventional clinicopathological variables. This indicates its potential as a complementary tool for risk stratification. In addition, the risk score showed strong correlations with immune cell infiltration and immune checkpoint gene expression, with high-risk patients exhibiting a more inflamed but potentially immunosuppressive tumor microenvironment. These findings suggest that high-risk patients may derive greater benefit from ICB therapy, a hypothesis that warrants further investigation in clinical trials. Indeed, recent studies have highlighted the potential of combining senescence-targeting agents with immunotherapy to overcome resistance and enhance therapeutic efficacy [37, 38]. Future studies should explore whether the high‑risk group is associated with T cell exhaustion and M2‑polarized macrophage phenotypes, which would further mechanistically link the CSRG signature to immunosuppression.

Among the 15 prognostic CSRGs, we identified EEF1E1 as a particularly intriguing candidate due to its specific enrichment in malignant hepatocytes at single-cell resolution. EEF1E1, also known as eukaryotic translation elongation factor 1 epsilon 1, has been implicated in aminoacyl-tRNA biosynthesis and cellular stress responses [39–41]. While its role in cancer remains incompletely characterized, emerging evidence suggests that EEF1E1 may contribute to tumor progression through mechanisms involving metabolic reprogramming and immune modulation [42]. Our validation studies confirmed that EEF1E1 is significantly upregulated in LIHC tumor tissues at both the mRNA and protein levels, and its high expression correlates with advanced histological grade and pathological stage. Moreover, EEF1E1 expression showed significant positive correlations with infiltration of multiple immune cell types, including T cells and macrophages, further supporting its potential involvement in shaping the immune landscape. Functional experiments demonstrated that EEF1E1 knockdown markedly suppressed the migratory and invasive capacities of LIHC cells, establishing EEF1E1 as a functionally relevant gene contributing to tumor aggressiveness. However, direct evidence linking EEF1E1 to cellular senescence or SASP is still lacking, and further mechanistic studies are required to confirm whether its pro‑tumorigenic effects are indeed senescence‑dependent. These findings position EEF1E1 as a promising therapeutic target in LIHC, and future studies should explore the molecular mechanisms by which EEF1E1 promotes tumor progression and immune modulation.

Despite the strengths of our study, several limitations should be acknowledged. First, all analyses were retrospective and derived from publicly available datasets, which may be subject to inherent biases; prospective studies are needed to confirm the clinical utility of our risk signature. Second, the functional experiments were limited to in vitro assays; in vivo studies using orthotopic mouse models and metastasis assays are required to further establish the role of EEF1E1 in tumor progression. Third, our single‑cell analysis was descriptive and relied on pre‑existing annotations from the TISCH database; independent verification and more advanced analyses (e.g., pseudotime, cell‑cell communication) were not performed. We did not assess whether its pro‑migratory and pro‑invasive effects are mediated through canonical senescence pathways (e.g., p16/p21, SASP factors such as IL‑6/IL‑8). Nor did we examine its impact on immune checkpoint gene expression. Addressing these mechanistic questions will be essential to establish a direct link between EEF1E1, senescence, and immune modulation. Finally, while our CSRG set was derived from four core GO terms that define the senescence process and its regulation, we acknowledge that cellular senescence involves additional molecular events (e.g., DNA damage response). Nevertheless, the prognostic signature derived from this focused set remained robust across independent cohorts, and future updates incorporating emerging senescence‑related databases may further refine the gene set.

In conclusion, this study provides a comprehensive characterization of CSRGs in LIHC through integrative bulk and single‑cell RNA‑seq analyses. We identified two distinct molecular subtypes with divergent immune phenotypes and developed a robust CSRGs‑based risk score that independently predicts patient outcomes. Notably, we pinpointed EEF1E1 as a key functional gene with oncogenic potential and a promising therapeutic target. Our findings advance the understanding of how cellular senescence shapes the tumor immune microenvironment in LIHC and may inform the development of personalized therapeutic strategies. Future prospective studies and in vivo experiments are warranted to translate these findings into clinical practice. Nevertheless, the connection between EEF1E1 and the senescence phenotype requires direct experimental validation in future studies.

## Supplementary Information

Supplementary Material 1.

Supplementary Material 2.

## Data Availability

All data used in this study are publicly available. The bulk RNA‑seq and clinical data of the LIHC cohort were obtained from the TCGA portal (https://portal.gdc.cancer.gov/). The external validation dataset was downloaded from the ICGC Data Portal (https://dcc.icgc.org/). The single‑cell RNA‑seq dataset GSE166635 was accessed through the TISCH database (http://tisch.comp-genomics.org/). The cancer‑immunity cycle activity analysis was performed using the TIP database (http://biocc.hrbmu.edu.cn/TIP/index.jsp). Cellular senescence‑related gene sets were retrieved from the GO database using the following accession terms: GO:0090398 (cellular senescence), GO:2000772 (regulation of cellular senescence), GO:2000773 (negative regulation of cellular senescence), and GO:2000774 (positive regulation of cellular senescence). Additional gene sets were obtained from the Molecular Signatures Database (MSigDB, https://www.gsea-msigdb.org/gsea/msigdb/). The GEPIA database (http://gepia.cancer-pku.cn/) and the HPA (https://www.proteinatlas.org/) were used for expression validation. All other data generated or analyzed during this study are included in this published article.
