# Supplementary material for: Integration of bulk and single-cell RNA-seq data identifies a cellular senescence-related prognostic signature in liver hepatocellular carcinoma
Source: Discov Oncol. 2026 Jun 10;17:1187. doi: 10.1007/s12672-026-05440-z (PMC13478048; doi:10.1007/s12672-026-05440-z)
Supplement: Supplementary file 1 [file 12672_2026_5440_MOESM1_ESM.pdf]

Integration of bulk and single-cell RNA-seq data identifies a cellular senescence-related prognostic signature in hepatocellular carcinoma

Supplementary Material

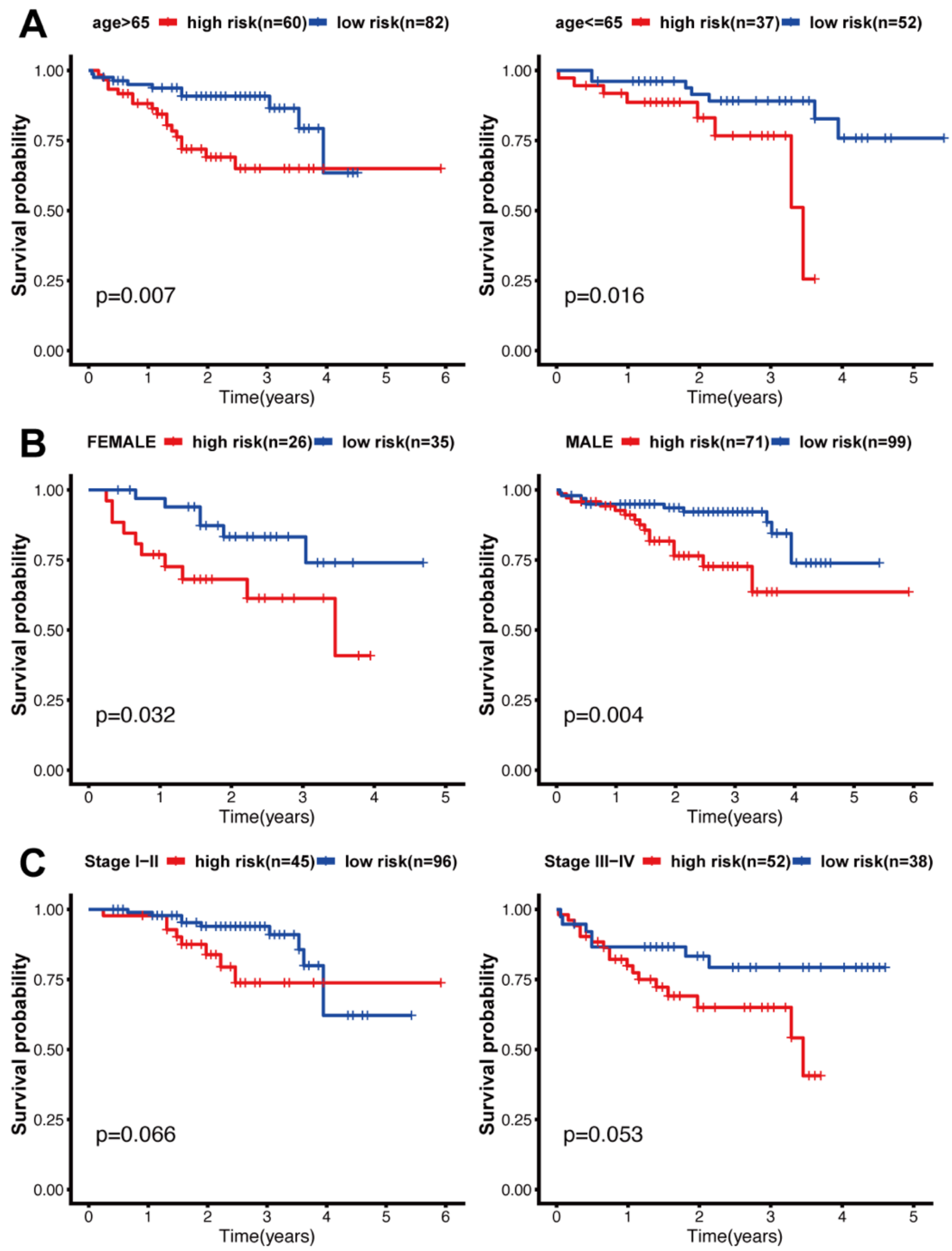

Figure S1

Figure S1. Subgroup survival analyses based on the CSRGs-derived risk score in the ICGC cohort.

(A–C) Subgroup survival analyses stratified by clinicopathological features in the ICGC dataset. In each subgroup, patients in the low-risk group consistently demonstrated superior survival outcomes compared with those in the high-risk group, including across different (A) Age, (B) Gender, (J) pathological stages, and (K)histological grades.

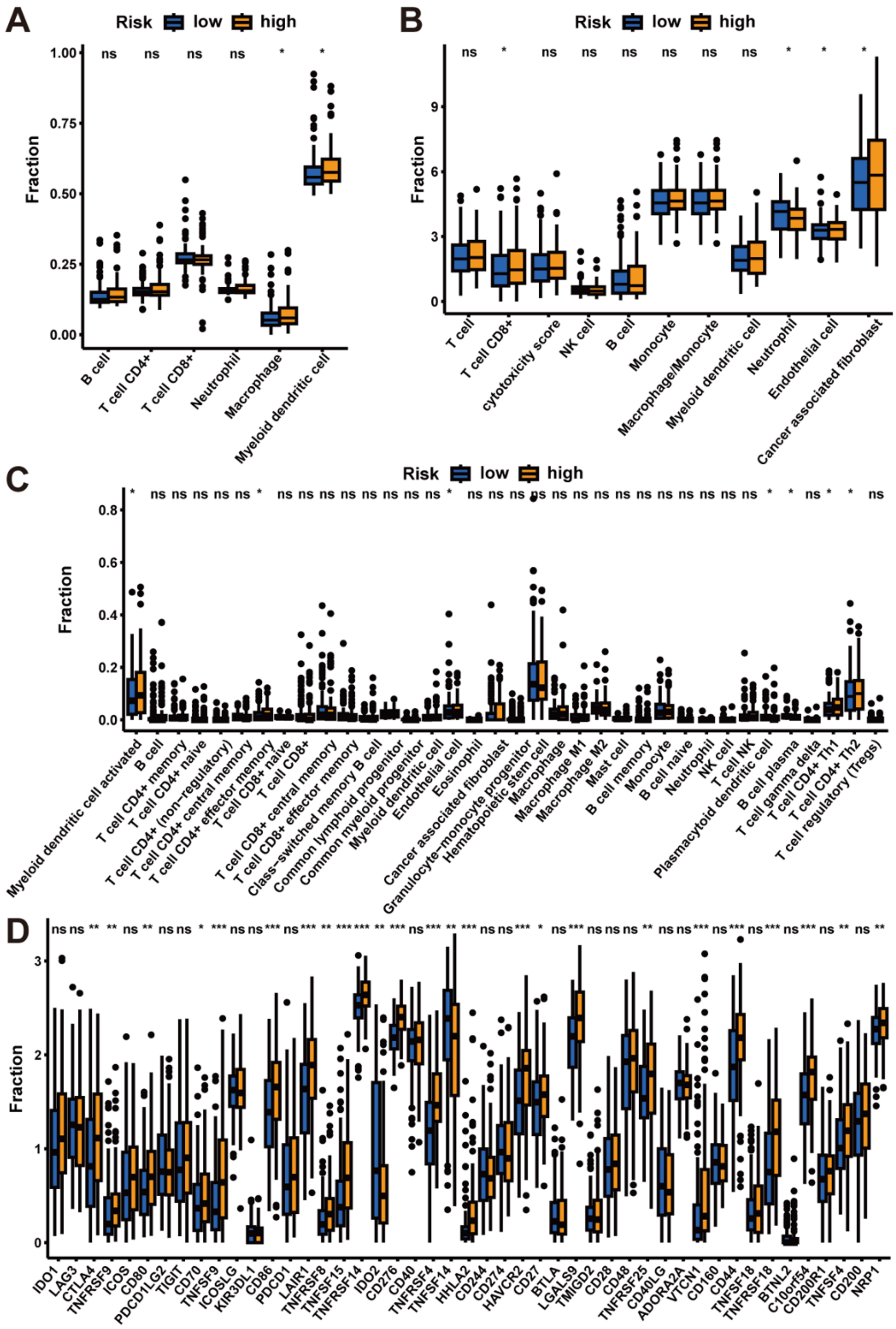

Figure S2

Figure S2. Immune landscape between high- and low-risk groups in the ICGC cohort.

(A–C) Bar plots showing the infiltration abundance of diverse immune cell types estimated by (A) TIMER, (B) MCP-counter, and (C) xCell algorithms in the ICGC cohort.

(D) Expression levels of canonical immune checkpoint genes in high-risk versus low-risk groups.

(*$P < 0.05$, **$P < 0.01$, ***$P < 0.001$)

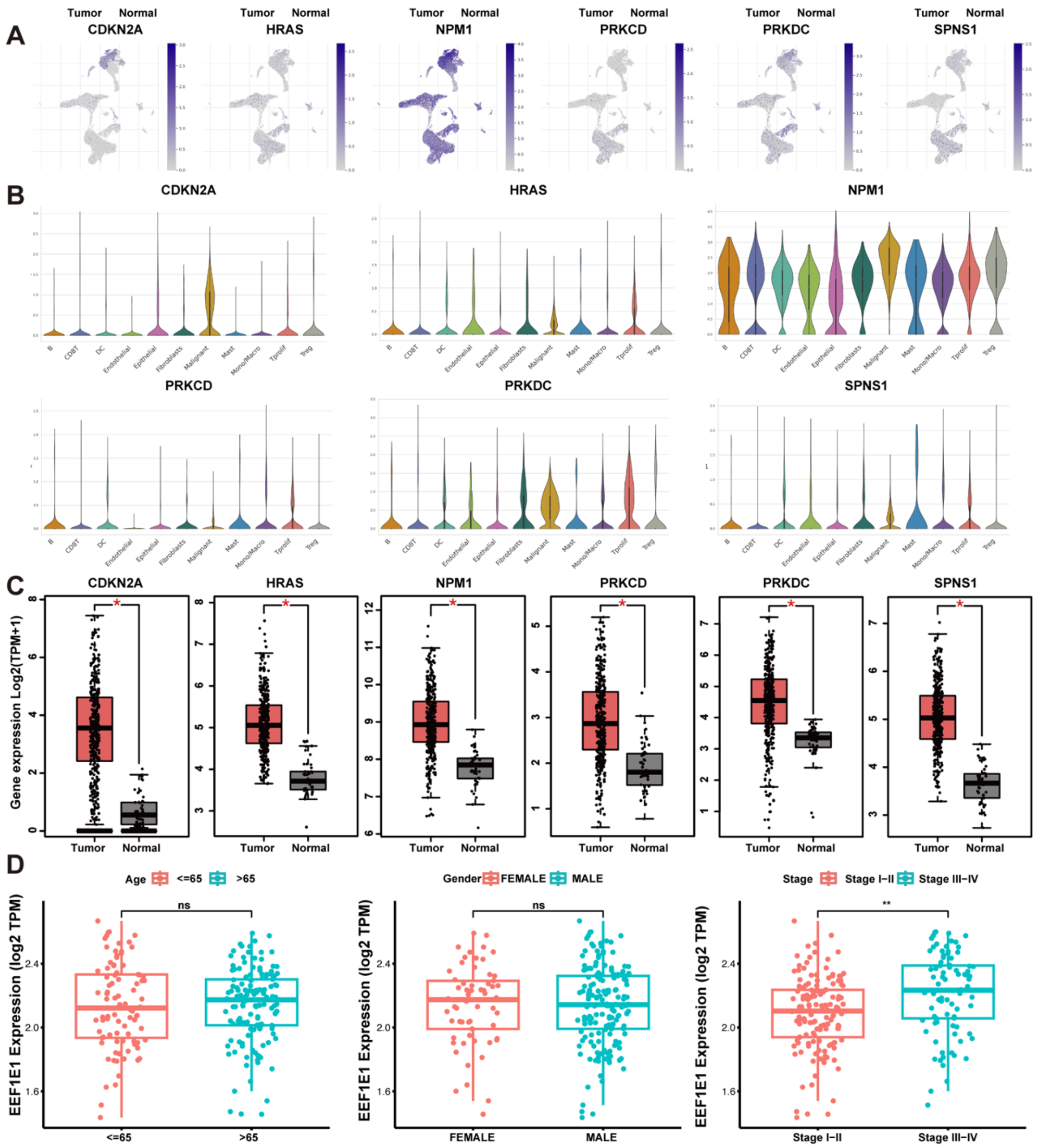

Figure S3

Figure S3. Single-cell expression patterns of the remaining six CSRGs in the LIHC tumor microenvironment.

(A) UMAP plots showing the expression distribution of the six prognostic CSRGs—CDKN2A, HRAS, NPM1, PRKCD, PRKDC, and SPNS1—projected onto the same dimensional reduction space.

(B) Violin plots showing the expression distribution of CDKN2A, HRAS, NPM1, PRKCD, PRKDC, and SPNS1 across distinct cell types in the GSE166635 single-cell dataset.

(C) Box plots showing the expression levels of CDKN2A, HRAS, NPM1, PRKCD, PRKDC, and SPNS1 in LIHC tumor tissues compared with adjacent normal tissues.

(D) The association between EEF1E1 expression and clinical pathological features in the ICGC dataset.

(*P < 0.05, **P < 0.01, ***P < 0.001)

Table S1. Genes and corresponding coefficients in the LASSO-based prognostic risk model.

Gene	Coef
HRAS	0.16107721
SPNS1	0.50329191
CDKN2A	0.04002537
PRKCD	0.08482043
NPM1	0.19099717
PRKDC	0.07141221
EEF1E1	0.16319351
