# Supplementary material for: Integration of bulk and single-cell RNA-seq data identifies a cellular senescence-related prognostic signature in liver hepatocellular carcinoma
Source: Discov Oncol. 2026 Jun 10;17:1187. doi: 10.1007/s12672-026-05440-z (PMC13478048; doi:10.1007/s12672-026-05440-z)

Figure 7C

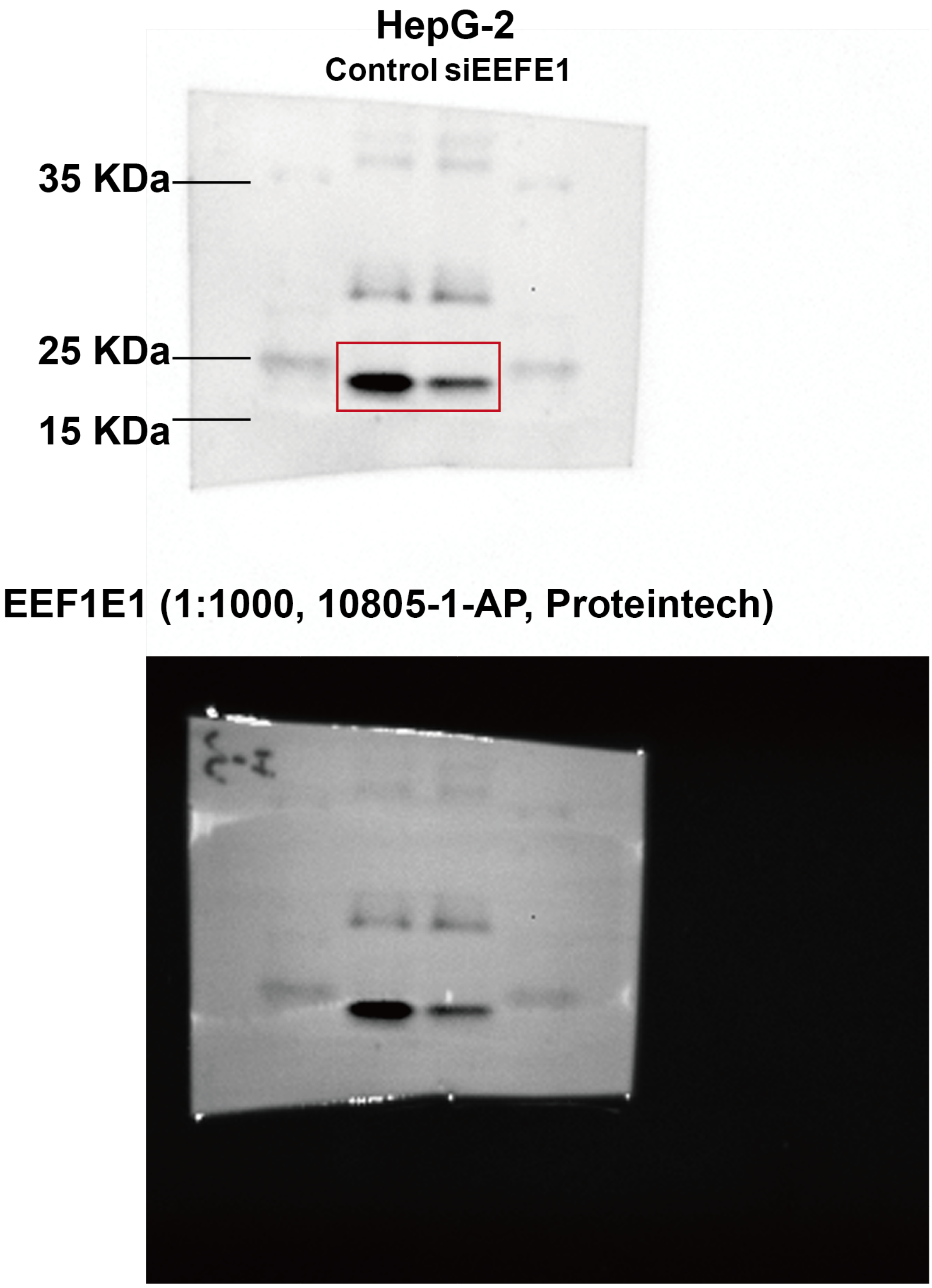
HepG-2
Control siEEFE1
35 KDa
25 KDa
15 KDa
EEF1E1 (1:1000, 10805-1-AP, Proteintech)

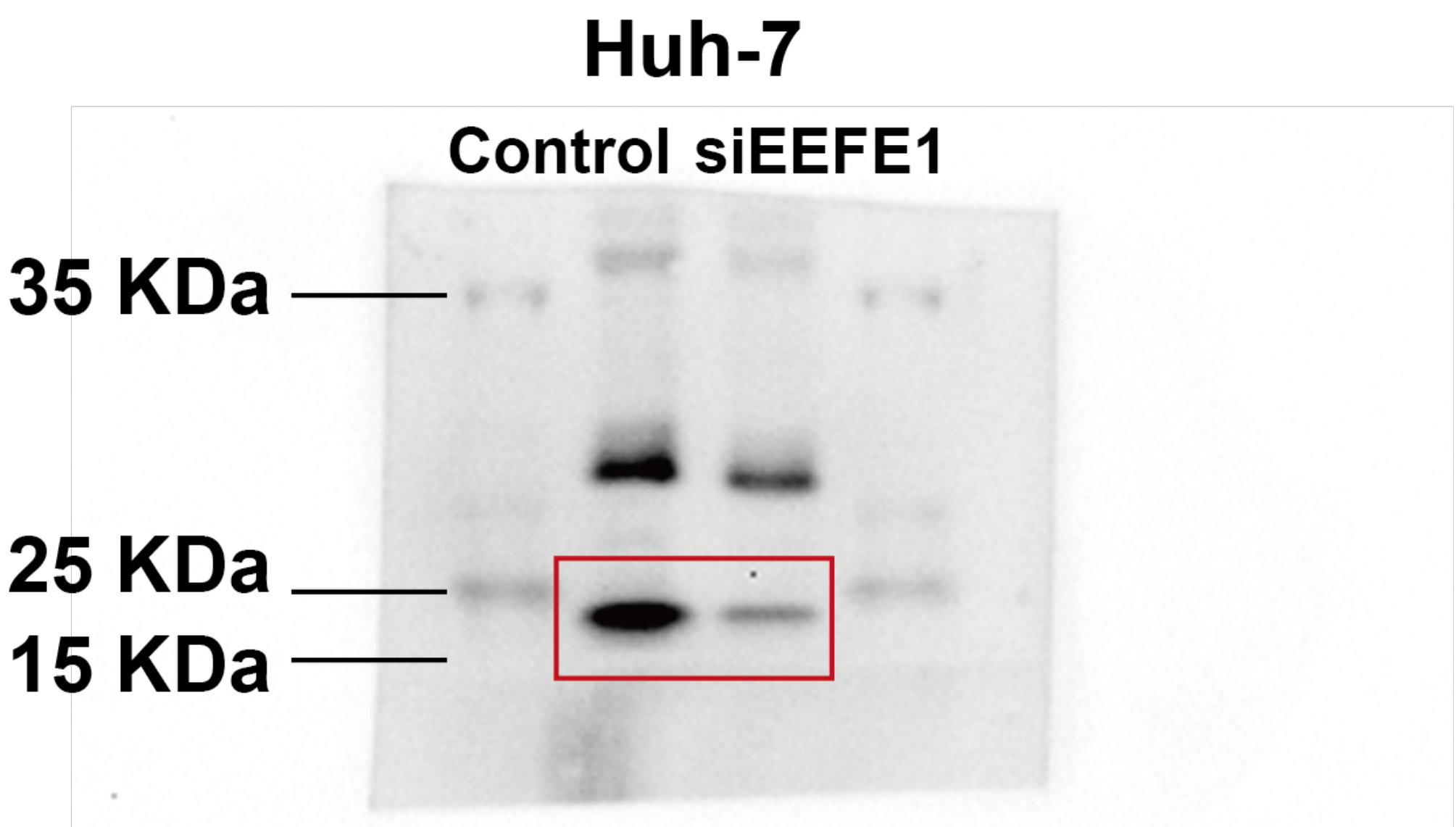

EEF1E1 (1:1000, 10805-1-AP, Proteintech)

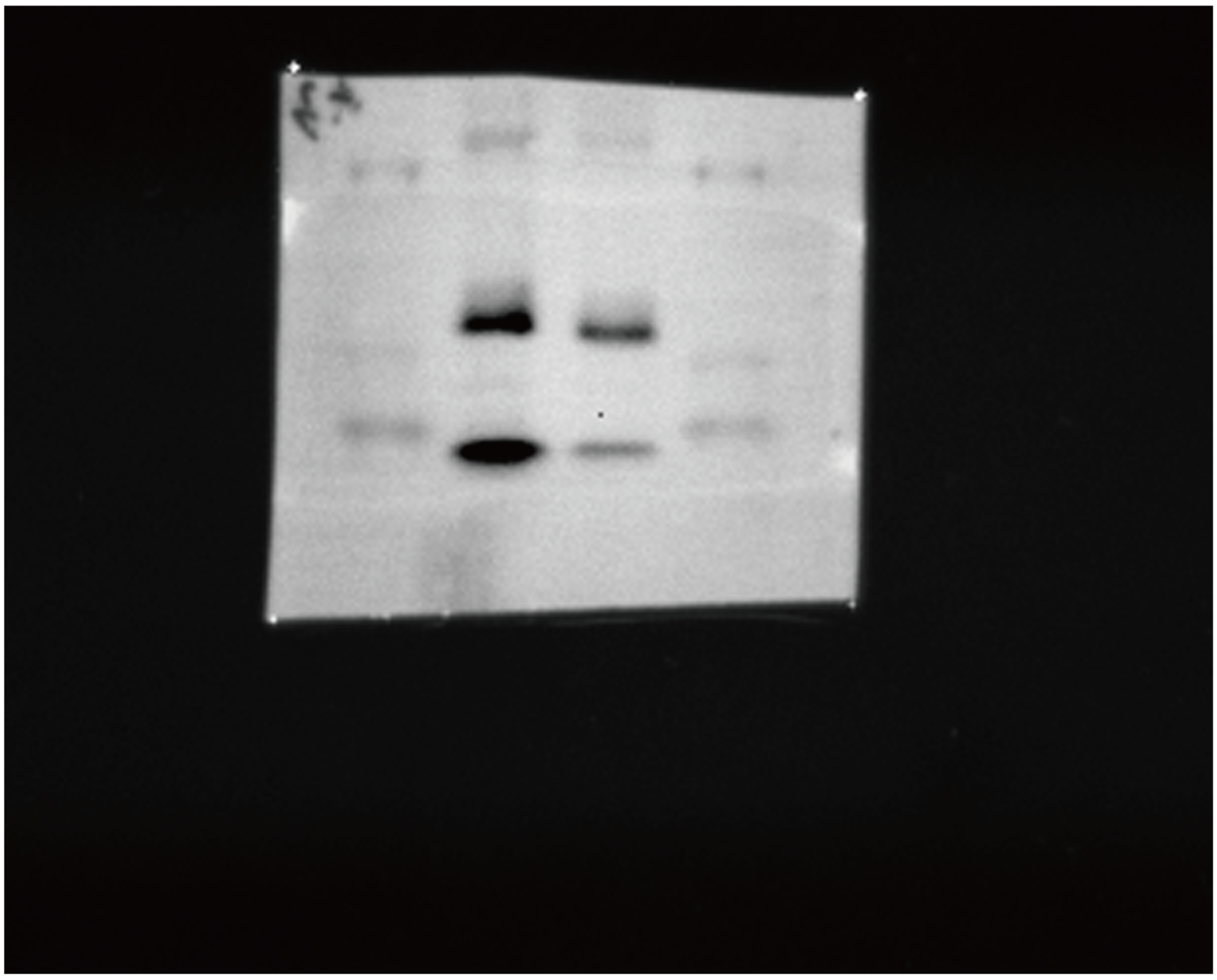

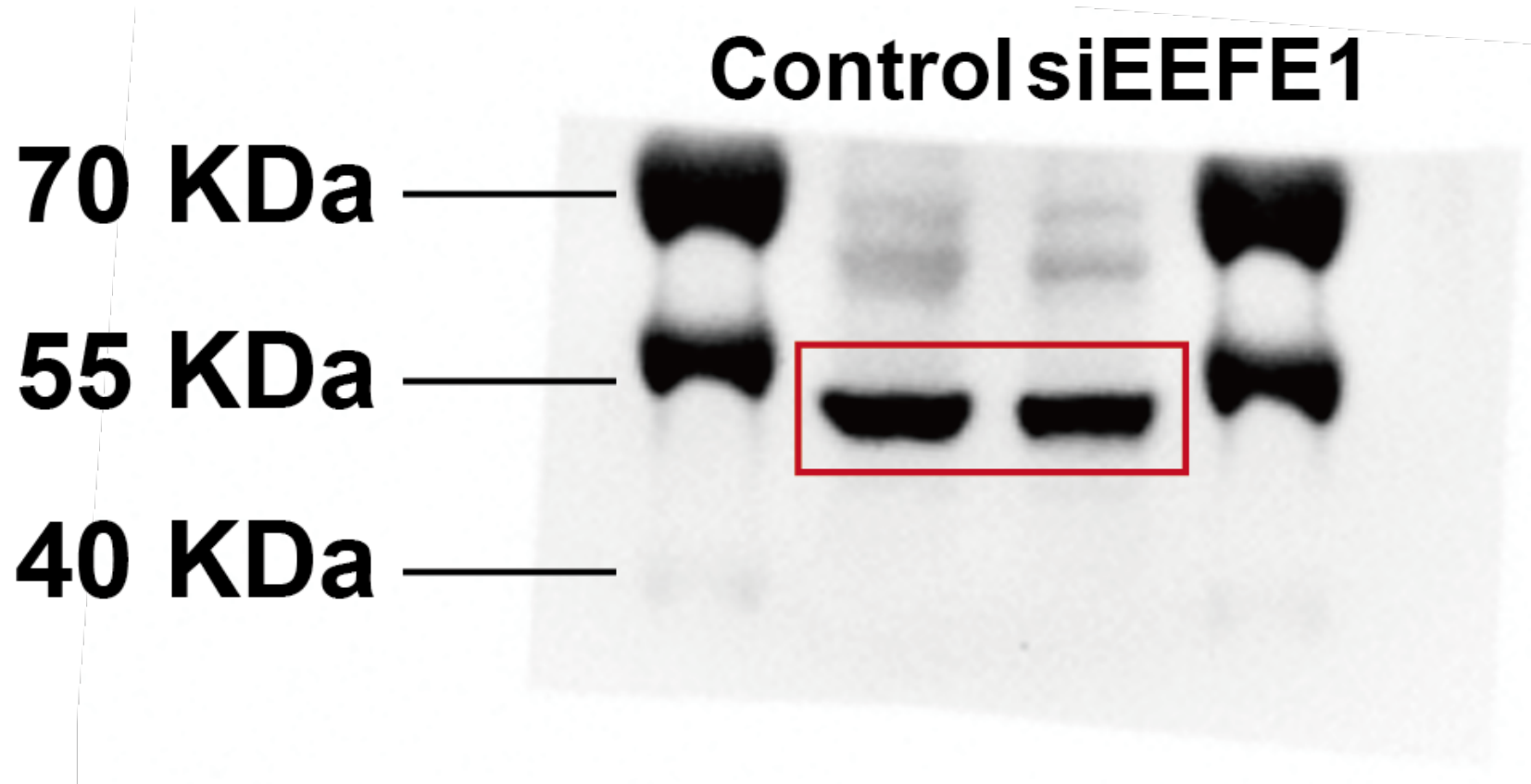

Tubulin (1:4000, AF1216, Beyotime)

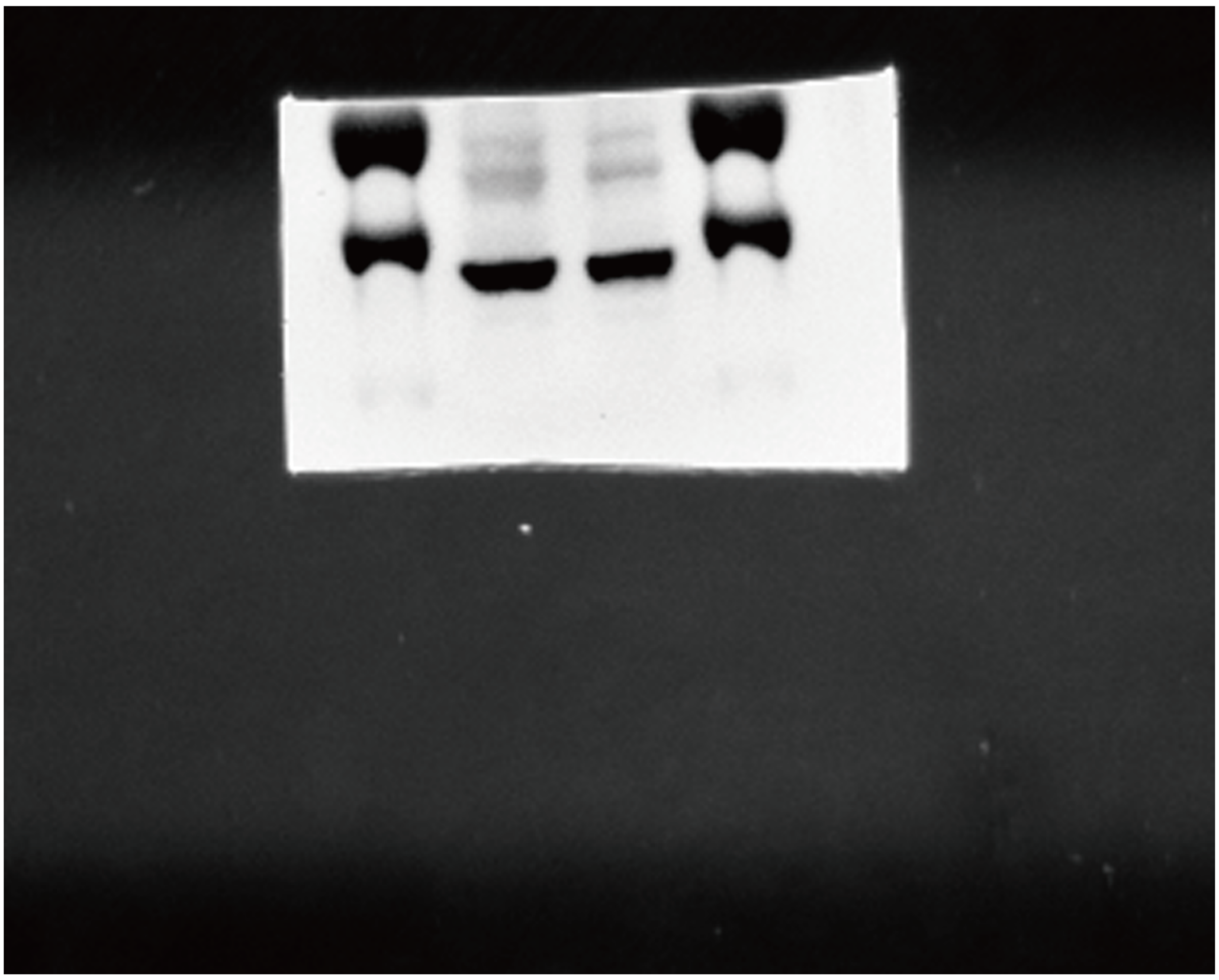

Supplement: Supplementary file 2 [file 12672_2026_5440_MOESM2_ESM.pdf]
